# Falsified Medicines Directive in a Secondary Care Environment—Impact on Supply Chain

**DOI:** 10.3390/ijerph19063276

**Published:** 2022-03-10

**Authors:** Piotr Merks, Urszula Religioni, Nuno Pinto de Castro, Anna Augustynowicz, Katarzyna Plagens-Rotman, David Brindley, Anna Kowalczuk, Justyna Kaźmierczak, Agnieszka Neumann-Podczaska, Eliza Blicharska, Katarina Fehir Sola, Martin J. Hug, Klaudiusz Gajewski, Paweł Piątkiewicz

**Affiliations:** 1 Faculty of Medicine, Collegium Medicum, Cardinal Stefan Wyszyński University, 01-938 Warsaw, Poland; piotrmerks@googlemail.com; 2 School of Public Health, Centre of Postgraduate Medical Education of Warsaw, 01-826 Warsaw, Poland; aaugustynowicz@poczta.onet.pl; 3 Open University Business School, Walton Hall, Kents Hill, Milton Keynes MK7 6AA, UK; nuno.pintodecastro@gmail.com; 4 Department of Economics of Health and Medical Law, Medical University of Warsaw, 02-091 Warsaw, Poland; 5 Institute of Health Sciences, Hipolit Cegielski State University of Applied Sciences, 62-200 Gniezno, Poland; plagens.rotman@gmail.com; 6 UCL Centre for the Advancement of Sustainable Medical Innovation (CASMI), The University of Oxford, Oxford OX2-0JB, UK; dave@davebrindley.com; 7 National Institute of Medicines, 00-725 Warsaw, Poland; apkowalczuk@gmail.com; 8 Zdrowit sp. z o.o., Pharmacy Chain, 41-940 Piekary Śląskie, Poland; j.kazmierczak@aptekizdrowit.pl; 9 Chair and Department of Palliative Medicine, Poznan University of Medical Sciences, 61-245 Poznan, Poland; ar-n@wp.pl; 10 Department of Analytical Chemistry, Medical University of Lublin, Chodźki 4a, 20-093 Lublin, Poland; elizablicharska@umlub.pl; 11 Pharmacy of Bjelovar, Petra Preradovića4, 43000 Bjelovar, Croatia; kfsola@gmail.com; 12 Medical Center–University of Freiburg, 79085 Freiburg, Germany; martin.hug@uniklinik-freiburg.de; 13 Imed Poland Sp. z o.o., 02-819 Warsaw, Poland; k.gajewski@zzpf.org.pl; 14 Department of Ophthalmology, Medical University of Warsaw, 02-091 Warsaw, Poland; piatkiewicz@op.pl

**Keywords:** falsified medicines directive, delegated regulation, supply chain, hospital

## Abstract

The Falsified Medicines Directive (FMD) and the Delegated Regulation (DR) impact the pharmaceutical supply chain. Ahead of the deadline for implementation, in February 2019, every entity of the supply chain had to adapt its operations to the regulatory requirements to be compliant with the directive. This paper analyzes the supply chain of a hospital pharmacy and the impact of the FMD implementation. Furthermore, a cost analysis was performed demonstrating that the FMD increases expenditure in the secondary care environment dispensing operations. Governments should be aware that this regulation will certainly impact public healthcare institutions in the long term.

## 1. Introduction

Pharmaceutical products represent a significant expenditure for the healthcare system of any country; for example, in Poland they represent about 10% of public spending for the healthcare system [1]. The awareness of the risk of using falsified medicinal products for patients is of vital relevance around the world. Supply chain management has been given little attention; optimization and efficiency are of great importance to minimize healthcare expenditures [2,3,4].

Analysis of the healthcare supply chain is quite common in the literature [5]. Other authors introduced models to generate value to the healthcare supply chain [6]. The healthcare supply chain cannot be considered linear or as a simple manufacturing supply chain, but it needs to be understood that services, products, knowledge, and regulation work together to protect the patient’s final beneficiary. Therefore, it can be considered a supply chain in that the information flows through the various intermediat stations.

Pharmaceutical products deserve special consideration in society, especially the institutions that dispense these products, which need to be aware of inventory control and the impact of new regulations on their operations. Medicines are developed, manufactured, and distributed according to strict regulatory requirements. Medicines account for a large part of health expenditure, and the increase of pharmaceutical costs and the arrival of innovative medicines will impact Gross Domestic Product (GDP), as in some countries the costs will be higher than GDP growth [7]. Consequently, optimization of the supply chain is of the utmost importance for healthcare institutions. The concept of value chain analysis allows verification of the full range of activities that generate a service or product’s delivery to achieve a competitive advantage and compete in the current marketplace [8].

The concept of value chains as decision-support tools was added to Porter’s competitive strategies paradigm as early as 1979. Activities within an organization add value to the service and products that the company produces, and all of these activities should be run at the optimum level if the organization is to gain any real competitive advantage. The activities could be split into two categories: primary activities and support activities [8]. Primary activities in the supply chain include elements such as the receiving of goods, warehouse operations, outbound, some simple elements of marketing and sales, and services related to the above, e.g., customer service.

Supporting activities mean being involved in the whole process and not only performing their basic elements. This includes, above all, increasing the effectiveness of the basic activities, applying feasibility indicators to them and ensuring their high performance. Another element of support is the analysis of the current situation related to the combined elements such as purchasing structure, infrastructure management, human resources management, technological development, and to deal with the optimization of these elements.

The European Union published the Falsified Medicines Directive (FMD), introducing a safety feature on the packages of drugs, ensuring the verification of a drug’s authenticity before supplying it to the patient [9].

The details of implementing a Europe-wide system for authentication of medicines is included in the Delegated Regulation (DR), published in February 2016. The deadline for DR implementation was 9 February 2019, with the authentication systems required to be operational and running before this deadline [10].

The FMD impacts the entire pharmaceutical supply chain [11]. The aim of the paper is an evaluation of the impact of this directive in the secondary care supply chain and the optimization of hospital pharmacy dispensing operations to be compliant with the requirements of the DR. Basically, our intention is to show the real costs of introducing FMD to the end user (hospital pharmacy). Our article presents three scenarios for the implementation of the directive, as well as the short-term and long-term cost approaches.

## 2. Impact of the FMD on the Pharmaceutical Supply Chain

The pharmaceutical supply chain involves a series of intermediate stations, from manufacturers and wholesalers, to healthcare institutions, pharmacies, and patients.

The FMD and respective DR impact every intervenient of this supply chain, especially in terms of the regulatory compliance that needs to be met in the nearest future [10].

### 2.1. Manufacturers

The main changes for pharmaceutical manufacturers are the new labelling regulatory requirements and adaptability of the production lines [12]. Medicinal products need to bear a unique identifier, a 2D bar code matrix on each medicine package compliant with ISO 16022 [10,13].

The unique identifier generated by the pharmaceutical industry needs to be reported to the European hub that identifies the code with a market authorization holding and reports the unique identifier to a national repository system that stores it until the moment of decommissioning is performed by the end user, who verifies the authenticity of the product through a scanning dispensing operation. Furthermore, each medicine package needs to contain an anti-tampering device. Manufacturers that perform wholesale activities and supply directly to healthcare institutions also need to verify and decommission medicinal products; further details are presented in Table 1 [10].

### 2.2. Wholesalers

Wholesalers also need to implement new operations under the FMD. They need to verify medicinal products returned by other parties (community and hospital pharmacy, wholesalers, other organizations that supply medicinal products) and medicinal products supplied by wholesalers who are not the market authorization holder (MAH). Depending on what type of operations wholesalers have, if they supply only wholesalers, if they receive products only from manufacturers, it changes the verification and decommission operations they need to perform.

There is no need to verify the authenticity if the medicinal product changes ownership but remains in the physical possession of the same wholesaler or the distribution of drugs is between a wholesaler’s own warehouses [10].

Wholesalers are responsible for decommissioning the unique identifier through the scanning of the 2D matrix bar code, removing the unique identifier from the national repository system. Decommission can be performed for several reasons. Table 1 reveals more details.

### 2.3. Healthcare Institutions–Community and Hospital Pharmacies

Persons authorized to supply medicinal products to the public need to decommission the unique identifier at the time of supplying it to the public; this can be accommodated easily in a community pharmacy perspective. The DR gives the possibility for hospitals to verify or decommission products in their internal supply chain, so this generates some options for them depending on the institutions [10,14].

Dispensing part of a pack also impacts dispensing operations, so healthcare professionals need to decommission the package before opening it.

## 3. Other Implications of the FMD That Affect All the Intervenients of the Pharmaceutical Supply Chain

### 3.1. Verification vs. Decommission

A medicinal product can be verified or decommissioned through an authentication system. The verification process allows the serial number of the package to be queried in a national repository system and, thus, the authenticity of the product can be verified. Verification can be performed several times. A decommission scanning process removes the code from the national database. This ultimately confirms that the product was dispensed and used [10].

### 3.2. Products Included in the DR

The majority of drugs that bear the safety features are prescription-only medicines (POM). Non-prescription medications are excluded. Annex I and II of the DR refer to medications that do not need to bear the safety features and to this end one over-the-counter (OTC) product that needs to bear the safety features, respectively. Also, member states can adapt the respective lists if they wish to include or exclude medicinal products that are at risk of being counterfeited; Annex III and IV serve that purpose [10].

So, manufacturers who depend on the product portfolio that they have do not need to adapt all their products to bear the safety features required by the FMD. Wholesalers and healthcare institutions do not need to perform verification and/or decommission of medicinal products that do not bear the safety features. Therefore, this knowledge can ultimately influence the efficiency of the supply chain.

### 3.3. The Tendays’ Rule

The DR introduces another important aspect that can impact the distribution and, especially, the returns for all of the supply chain: the 10-day rule from Article 13, which pertains to reverting the status of the unique identifier. From the moment the decommission is performed, the organization has 10 days to revert the unique identifier’s status [10,15,16].

If that period elapses, the medicinal product can only be used in the respective physical location where it was decommissioned. In the case of wholesalers of the manufacturers decommissioning the products to supply some organizations, it is difficult to revert the status of the unique identifier from the moment the product is supplied, reach the organization, and decide to return it before the 10 days elapse. When manufacturers and wholesalers decommission medicinal products, possible returns from the organizations induced in article 23 of the DR is impossible.

This significantly impacts distribution in the entire supply chain. Furthermore, it can impact several organizations economically (Table 1).

## 4. The Pharmaceutical Supply Chain: A Hospital Perspective

This paper targets the hospital pharmacy supply chain and the impact of the FMD in the dispensing operations. While some of the work in this field relates to problems with FMD implementation or readiness for implementation [15,16,17,18], our work analyzes the actual supply chain and proposes a new supply chain under the FMD requirements. The case hospital pharmacy supply chain is based in the Austrian Tirol region. The hospital pharmacy supplies all regional hospitals in the Austrian Tirol, 16 hospitals, so it works as a centralized hub for the region. The hospitals that are supplied do not have a hospital pharmacy [19].

It dispenses a total of 2.25 million packages a year of the 112,000 orders received. The group of hospitals has a capacity of 4898 beds; in terms of logistics, the implication of the FMD for this type of operation is significant [19]. The FMD is a good opportunity to look at the supply chain and adapt it to a new reality and possibly optimize it.

**Figure 1:**
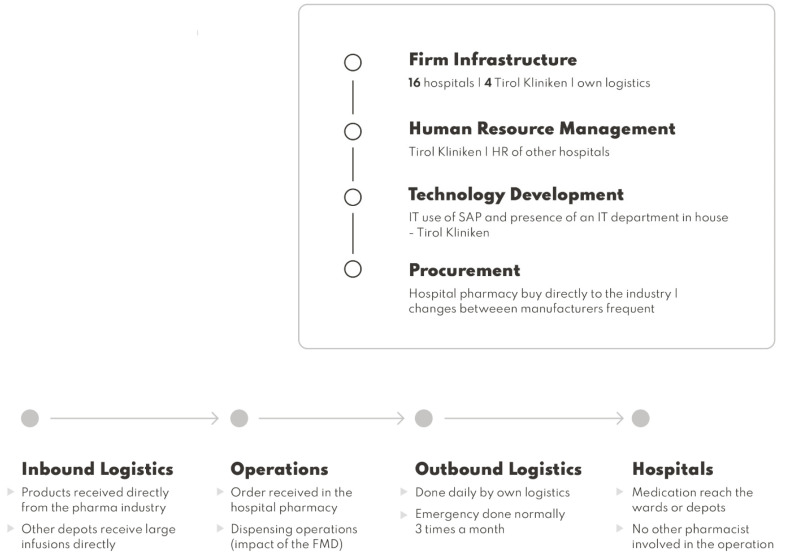
Porter’s value chain analysis of the present supply chain of the case hospital.

**Figure 2:**
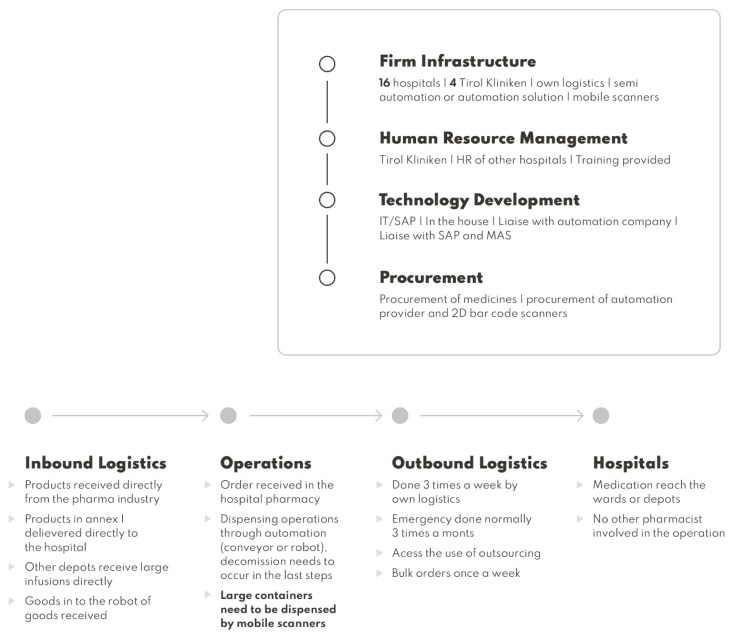
Porter’s value supply chain analysis of the case hospital in a FMD environment.

Using Porter’s value chain analysis, some inefficiencies can be resolved [8] (Figure 1). The implementation of the provisions of the FMD significantly increases the value of the supply chain, which is understood as the activity of the organization aimed at providing the end user with the product that he expects. This allows for the delivery of safe medicinal products to patients, which will be presented later in the article, in Figure 2.

The case hospital pharmacy supports a total number of 16 hospitals in the Tirol region, so in terms of infrastructure there is a need to support the delivery activities. Outsourcing the supply chain can lead to more expenses. A public procurement exercise can be made with delivery providers to possibly reduce costs.

Due to implementation of the FMD, there is a need to adapt the new bar code readers of the hospital pharmacy to fall on the infrastructure responsibilities to procure the best viable options. Eventually, if the hospital selects automation as a possibility, negotiations need to occur with the respective providers.

Human resources need to deal with the possible costs of hiring new staff and the necessary training if they are not qualified to dispense medication. Other studies refer to the possibility of increasing the number of staff because of the implementation of the FMD [19].

IT needs to adapt to FMD implementation since the medicines authentication system is running as an IT solution in the hospital’s actual software. Eventually, if automation is installed, IT will be involved, since this can be a possible solution to reduce the hiring of new employees [19].

Procurement is done by the hospital pharmacy. If eventually some of the manufacturers introduce aggregated codes, medicinal product procurement of those products needs to be prioritized because it will benefit dispensing operations [20].

In terms of primary activities, the inbound logistics is done by the manufacturers that supply directly to the case hospital pharmacy. Sometimes large infusions are supplied directly to the other remaining hospitals.

In terms of operations, orders are received in the hospital pharmacy by different wards and other hospitals in the group. The collection and preparation of the order starts with manual dispensing. The collection is done manually and some scanning is involved just for inventory management purposes, so not all of the products dispensed are scanned. Orders, such as for infusions and antibiotics, are then sent to wards by totes or by bulk in large packages.

Outbound logistics is done to 16 hospitals in the region; medication is delivered daily by their own means. Outsourcing can occur if needed. Emergency medication can be delivered when needed. Hospitals dispense the medication to the patients in the wards.

## 5. The Impact of FMD Implementation on the Supply Chain of a Hospital

A proposed supply chain is detailed with the relevant impact of the implementation of the FMD in operations. The support activities, regarding infrastructure for FMD implementation, and the need for extra human resources to implement automation, can be a solution and the procurement of an automated solution can be considered. Normally, companies provide automation provide scanners for the rest of the operations that cannot be done through automation. If not, procurement of 2D bar code scanners needs to be made [21,22].

Regarding human resources, if the option of automation is not pursued a recruitment process needs to be planned, and if automation is pursued, a training is normally done by the company that supplies the solution, but a possible plan for training needs to be implemented.

The IT department possibly has to adapt the dispensing software with the final interfaces of verification and decommission that are going to be delivered by the blueprint provider. If automation is implemented normally, new software comes with the solution that needs to be implemented and integrated with the dispensing software, so IT needs to liaise with the automation company if any issues arise with the dispensing operations.

The procurement of medicines can be done directly through the pharmaceutical industry that selects the products containing aggregation to reduce operational dispensing time [20,21]. Delivery to other hospitals can re-think other options that can be pursued by accessing the costs of outsourcing or through tender processes to evaluate if there are more inexpensive ways of delivering to reduce the impact of expenditures in daily deliveries. Public tender processes for automation and possibly new scanners are needed.

The primary activities have the biggest impact in terms of adapting to the FMD regulatory requirements.

The inbound logistics continue to be done by the manufacturers. Nevertheless, change can be adapted to achieve better efficiency. Procurement can be done centrally but the dispatch of certain drugs can be done directly to the specific hospitals. Instead of being received by the case hospital centrally and then dispatched, this can transfer delivery costs to the supplier. Large infusions and medications that are recurrently used can be delivered from the industry, bypassing especially the ones included in Annex I of the DR. Also, manufacturers deliver medicinal products with the respective unique identifier in each medicine package. If automation is an option, inbound goods to the robot need to occur at this point.

In terms of operations, orders are going to be received in the same way; if automation or semi-automatic solutions are pursued, the operator needs to select the order and dispense it through the system. Decommission needs to be performed on goods outside of the solution. Other possible solutions for the dispensing operations are mobile scanners because of the pharmacy’s infrastructure; the decommission of medicinal products at the dispensary eases the workflow and the distribution of medicinal products for the internal hospital and external ones [23].

To optimize the operations, there is a need to change the storage format; items that need to be authenticated should be close to the dispensary. The others included in Annex I of the DR can be stored in other locations so that the staff easily differentiate the products that need to be decommissioned or not.

Other regulatory implications for operating in a FMD environment are required. If a split pack is dispensed, the original container should not leave the dispensary until its entire content is used; the pharmacy cannot re-sell or supply other pharmacies with this product. For products sent to the wards and to external hospitals, the authentication can be done when the item is dispensed to the wards. Because this decommissions the serial code from the database, this needs to be the last step of the workflow. Manual authentication should also be performed in the last step by an accredited pharmacy technician or pharmacist [24]. Drugs manipulated in the cytotoxic clean room can also be authenticated in the dispensary before being distributed to this area.

Other recommendations in terms of operations achieved in other studies also impact the supply chain. A verification of the serial code can be done if necessary at any point of the supply chain, including in the workflow of the pharmacy. Medicines identified as falsified or recalled should be quarantined for inspection by suitably qualified professionals. Also, once a national medicines serial code repository is established and in operation, any medicine returned to the pharmacy, intended for re-use, should be verified. Authentication should be incorporated into departmental procedures. Incidents, when medicinal products leave a dispensary without authentication, should be classed as a dispensing error. If a medicine has been authenticated but is no longer required for the current dispensing process, there should be an option to return the unique identifier scanned to the national repository database [23]. Products that become non-compliant with FMD during the duration of the directive are considered in the system as unverifiable and their codes will cease to be active when the expiry dates are reached. It is the most economical solution because it is cost-free.

The outbound logistics can also be adapted. A better assessment of the resources, in terms of medicines, a hospital needs should to be made to understand if logistics can be changed. Large orders of electrolytes and high consumables can be delivered once a week by bulk, instead of being included in a daily order. The manufacturer can directly deliver medicinal products that are included in annex I of the DR to other hospitals. Orders should be done three times a week instead of daily to reduce the cost of logistic operations. The cost of possible outsourcing of logistics needs to be confirmed to investigate the impact on the overall costs of logistics. A secure supply chain to the wards and other hospitals under GDP is needed to be implemented to safeguard the authentication process [25].

Hospital wards deliver the drugs to the patients. Figure 2 summarizes the observations.

## 6. Financial Assessment and Impact of the Implementation of the FMD

Implementing the FMD in daily operations in the hospital environment has a financial impact on the organization.

Table 2 exposes a summary of possible scenarios of implementation. The data was collected from the case hospital and semi-automatic and automatic solutions from companies that supply the systems.

Scenario 1 is the implementation by increasing the number of employees. Several studies refer to the increase of operational dispensing time [19,20]. In the case of a hospital, there is a need to increase 3.5 FTE, which increases the hospital pharmacy’s expenditure in salaries.

Scenario 2 gives a semi-automatic solution and a slight increase in the number of employees. Implementing a conveyor belt with an image recognition system reduces the burden for implementation of the FMD since this solution can dispense 2000 packages/hour. The warehouses contain conveyor belts and at the end of the conveyor, there is an image recognition system incorporated with a scanner that recognizes the drug and scans the 2D bar code. The conveyor then diverts the drug to the specific token for delivery to the relevant ward or hospital [23]. Nevertheless, there is a need to increase the number of employees slightly since the semi-automatic solution still involved picking medicines from the shelve and placing them on the conveyor belt. The reduction of the number of mobile scanners is also reduced since the scanning operations are done through the image recognition system.

Scenario 3 describes the fully automated solution. By implementing this kind of solution, only one FTE is needed to implement the FMD in the hospital effectively. Automated package solutions operate with effectiveness [23]. The costs of hiring and training, and of procuring scanners, are reduced. The solution is the most expensive of the three scenarios, especially in the first year.

In Scenario 2 and Scenario 3, semi-automatic or automatic solutions are introduced. Additional costs related to these improvements such as installation, service, system maintenance, and IT work are included in the price of the equipment. Possible costs for the adaptation of the premises are assumed to be minor as the implementation can be done in pre-adapted places. From an economic point of view, all elements are parts of the hospital retrofit and their cost increases the value of the unit and will be depreciated over time—which will generally be even better reflected in the hospital accounts.

Regarding the three scenarios proposed in the paper Scenario 2 is the less expensive to implement, followed by Scenario 3 and Scenario 1, respectively. There is also the understanding that maintenance, updates and Moore’s law impacts the expenditures in scenarios 2 and 3 because of the semi-automatic and automatic solutions in place. The analysis showcases that the increase in expenditures continuously in years, especially for Scenario 1, is greatly dependent on employees. An on-off cost for hiring and training is also considered in each scenario. Also, there is a need to consider that employees have the right to holidays and sick leave that can affect the operations. Continuous professional development costs are not taken into account. Furthermore, the analysis also includes the procurement of the necessary hardware (mobile) for the hospital pharmacy since there is a need to scan and decommission each medical package dispensed.

Also, after 5 years of implementing Scenario 3, the solution is paid for and the expenditure is reduced as compared to Scenario 1.

If we take into account the cost of daily delivery per hospital a year, EUR13,900 per institution. The case hospital supply 16 institutions which costs EUR222,400 for deliveries. A reduction can be established if deliveries will be done three times a week only, it will cost EUR6,700 a year per institution (internal data from Innsbruck hospital), which will cost EUR107,200. It will reduce expenditure by EUR115,200, which can be re-located to the costs of implementation of the FMD. Through this solution Scenario 1 will cost EUR17,800, Scenario 2 cost EUR7,227.5 and Scenario 3 costs EUR426,085 for implementation in the first year.

### Limitations

In our work, we focus on serialization, but we do not deal with the issue of computerization and system connection in a broad context, which can be a challenge for many hospitals. Thus, the limitations of IT systems in the implementation of this type of solutions may be the direction of further research in this area.

## 7. Conclusions

This paper demonstrates the impact of the FMD on the pharmaceutical industry supply chain. Every single intervenient is affected by the regulation and, consequently, the operations that need to be introduced need to be FMD compliant.

The costs for implementation in a hospital environment could be very high since operations need to be modified and a changed management project needed to be introduced. Healthcare constraints in Europe are constant, and since the financial crisis in 2008 the majority of EU countries have suffered huge cuts.

Health departments responsible for implementing the FMD in public institutions will have to look at the cost to accommodate this directive in their public hospitals.

Automation or semi-automatic solutions can be implemented for hospitals that dispense large quantities of medicines each year. Centralized dispensing can also be implemented if needed.

## Figures and Tables

**Table 1:** Summary of the impact of the FMD in the pharmaceutical supply chain.

Manufacturers	Wholesalers	Healthcare Institutions (Community and Hospital Pharmacies)
Introduction of the unique identifier and anti-tampering device on medicine packages.	Understand the type of operations that the wholesaler has in place, what organisations are supplied, and from where they receive their products.	Decommission the unique identifier of the medicinal product before supplying it to the public.
Transfer encrypted data of the unique identifier to the EU Hub to be available in the national repository systems of respective member states of the MAH.	Verify medicinal products returned by other parties (community and hospital pharmacy, wholesalers, other organisations that supply medicinal products).	Hospitals have the possibility to decommission medicinal products in their internal supply chain depending on their workflow and selection.
Verify medicinal products returned by other parties (community and hospital pharmacy, wholesalers, other organisations that supply medicinal products).	Decommission medicinal products: distributed outside the European Union; for destruction (for example, recalled or expired products).	Workflow assessment is important to reduce expenditure and identify the best authentication points.
Decommission medicinal products: Distributed outside the European Union; For destruction (for example, recalled or expired products); Returned that cannot be re-sold (fridge products in some EU countries); Requested as a sample by competent authorities; Supplied to institutions listed in the article 23 of the Delegated Regulation (DR);	Returned that cannot be re-sold (fridge products in some EU countries); Requested as a sample by competent authorities; Supplied to institutions listed in the article 23 of the Delegated Regulation (DR).	
**Impact for all of the supply chain**		
Verification and decommission are different processes.		
Some products are excluded to bear the safety features required under the FMD, for: Manufacturers: there is no need to change labelling requirements or adapt the production lines, this is optional; Wholesalers and healthcare institutions: there is no need to perform verification or decommission of these products		
The impact of the 10-day rule: on operations; returns to the supplier; budget impact.		

**Table 2:** Financial analysis exposing different scenarios of the implementation of the FMD in the case hospital.

Scenario 1	Cost (€)	Scenario 2	Cost (€)	Scenario 3	Cost (€)
Manual dispensing + increasing FTE		Semi-automatic solution + increasing FTE		Automation solution + increase of FTE	
1 FTE salary/year 3.5 FTE salary/year	30,285 106,500	1 FTE salary/year 1.5 FTE Salary/year	30,285 454,275	1 FTE salary/year	30,285
1 Traditional scanner 6 Traditional scanners	500 3000	1 Mobile scanner 3 Mobile scanners	1500 4500	1 Mobile scanner 2 Mobile scanners	1500 3000
Cost of hiring 1FTE Cost of hiring 3.5 FTE	3000 10,500	Cost of hiring 1FTE Cost of hiring 1.5 FTE	3000 4500	Cost of hiring 1FTE	3000
Cost of training 1FTE Cost of training 3.5 FTE	2000 7000	Cost of training 1FTE Cost of training 1.5 FTE	2000 3000	Cost of training 1FTE	2000
		Conveyor belt + image recognition system	65,000	Robotic package dispensing solution	500,000
1 year	127,000		1,224,275		538,285
2 year	233,500		167,855		568,570
5 years	553,000		3,041,375		659,425
10 years	1,085,500		531,275		810,850

## Data Availability

All data are available from the corresponding authors.
